# Nutrient metabolism in regulating intestinal stem cell homeostasis

**DOI:** 10.1111/cpr.13602

**Published:** 2024-02-22

**Authors:** Ruicheng Shi, Bo Wang

**Affiliations:** ^1^ Department of Comparative Biosciences, College of Veterinary Medicine University of Illinois at Urbana‐Champaign Urbana Illinois USA; ^2^ Division of Nutritional Sciences, College of Agricultural, Consumer and Environmental Sciences University of Illinois at Urbana‐Champaign Urbana Illinois USA; ^3^ Cancer Center at Illinois University of Illinois at Urbana‐Champaign Urbana Illinois USA

## Abstract

Intestinal stem cells (ISCs) are known for their remarkable proliferative capacity, making them one of the most active cell populations in the body. However, a high turnover rate of intestinal epithelium raises the likelihood of dysregulated homeostasis, which is known to cause various diseases, including cancer. Maintaining precise control over the homeostasis of ISCs is crucial to preserve the intestinal epithelium's integrity during homeostasis or stressed conditions. Recent research has indicated that nutrients and metabolic pathways can extensively modulate the fate of ISCs. This review will explore recent findings concerning the influence of various nutrients, including lipids, carbohydrates, and vitamin D, on the delicate balance between ISC proliferation and differentiation.

## Graphical abstract

This review systematically describes the regulation of intestinal stem cell (ISC) homeostasis by metabolism, highlighting the role of feeding conditions, lipids, carbohydrates, vitamins and microbe metabolites in ISC proliferation, differentiation and tissue regeneration.

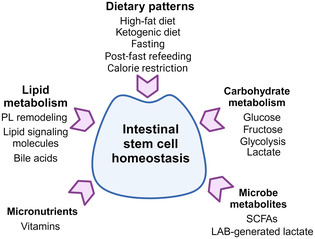

## 1 INTRODUCTION

Due to its constant exposure to mechanical pressure and luminal content during food digestion, the intestine poses a hazardous environment where intestinal epithelial cells undergo continuous damage and replenishment at an extraordinary rate. Intestinal stem cells (ISCs) and progenitor cells play a crucial role in maintaining this delicate balance through rapid self‐renew and differentiation.^1^ However, the determination of whether these cells should remain in a state of plasticity or undergo differentiation requires tight supervision and precise regulatory mechanisms. Any disruption in this intricate network can lead to the development of intestinal diseases, such as inflammatory bowel diseases and cancer.^2^, ^3^, ^4^, ^5^, ^6^ Therefore, a better understanding of the regulatory mechanisms that govern ISC fate decisions is crucial, as it is critical to developing potential solutions for future regenerative medicine.

Recent advances in tissue culture and single‐cell sequencing have significantly enhanced our understanding of ISC fate decisions.^7^, ^8^, ^9^ In addition to the well‐known signalling pathways that regulate ISC proliferation and differentiation, several metabolites have emerged as pivotal network ‘nodes’ tightly interconnected with extrinsic dietary cues. In this review, we provide a concise overview of previously well‐documented pathways, recent findings on key metabolites influencing ISC proliferation, and the intricate interplay between newly identified metabolites and established pathways.

## 2 THE INTESTINAL EPITHELIUM

In the small intestine, tubular microstructures invaginate through intervillous regions, forming finger‐like niches known as crypts.^1^, ^10^ The crypts are the home to ISCs and progenitor cells that maintain intestinal homeostasis and epithelial self‐renewal.^1^ ISCs reside in the crypt bottom, where they are protected from the mechanical pressure caused by digestion. ISCs come in contact with Paneth cells, which provide support and proliferation guidance through various signalling pathways, such as WNT, epidermal growth factor (EGF) and Hippo.^1^, ^11^ ISCs undergo division and give rise to daughter stem cells and progenitor cells. These progenitor cells move up into the transit‐amplifying zone, where they rapidly proliferate.^1^, ^12^ As they migrate from the crypts, the progenitor cells further differentiate into enterocytes, goblet, enteroendocrine, tuft and M cells while migrating from the crypts.^1^, ^10^ Paneth cells, on the other hand, move downwards and rendezvous with ISCs.^1^, ^11^ Meanwhile, other differentiated epithelial cells are constantly pushed upwards by newly born cells towards the villus tip, where they will eventually undergo apoptosis and detach from the villus through anoikis. In mice, the entire epithelial turnover cycle takes approximately 3–5 days to complete.^1^ To compensate for such high turnover rates, it is estimated that nearly 300 million new cells are generated to replenish the total epithelial cell pool.^1^, ^13^

## 3 IDENTITY OF ISCS

The ISCs were first described as crypt base columnar (CBC) cells in 1974 by Cheng and Leblond, which were later identified as leucine‐rich repeat‐containing G protein‐coupled receptor 5 (LGR5) positive cells.^14^, ^15^ *LGR5* encodes a seven‐transmembrane receptor of the secreted WNT agonist, R‐SPONDIN, which plays a crucial role in WNT signal transduction in ISCs.^16^ LGR5^+^ ISCs are intermingled with Paneth cells and divide daily, thus regarded as active stem cells. Several other markers, including OLFM4 and Prominin1/CD133, were later reported to mark LGR5^+^ ISCs as well.^17^, ^18^

Another ISC population has been described as quiescent, reserve, label‐retaining or + 4 ISCs. These ISCs are less proliferative under normal conditions but can be activated to proliferate and generate daughter cells in response to damage. Several markers have been identified to label these ISCs, including BMI1, HOPX, LRIG1 and mTERT.^19^, ^20^, ^21^, ^22^, ^23^ While ISCs with these markers share similar properties, such as radiation resistance and slow cycling, direct comparison reveals significant differences between ISCs labelled with different markers. For instance, the frequency and location along the intestinal tract of ISCs identified by these markers are distinct between these studies.^19^, ^20^, ^21^, ^22^, ^23^, ^24^, ^25^, ^26^ Therefore, their identity and relationship with CBC cells remains to be determined.

The single‐cell RNA sequencing (scRNA‐seq) technology has greatly facilitated the identification of new ISC sub‐populations. A recent study has profiled the regenerating mouse intestine after radiation damage using scRNA‐seq and identified a new Clusterin‐high expressing ISCs named revival stem cells (revSCs).^27^ These ISCs are extremely rare under normal conditions but activated to give rise to all major cell types in the intestine, including LGR5^+^ CBC cells during damage.

## 4 MAJOR SIGNALLING PATHWAYS REGULATING ISC HOMEOSTASIS

**FIGURE 1:**
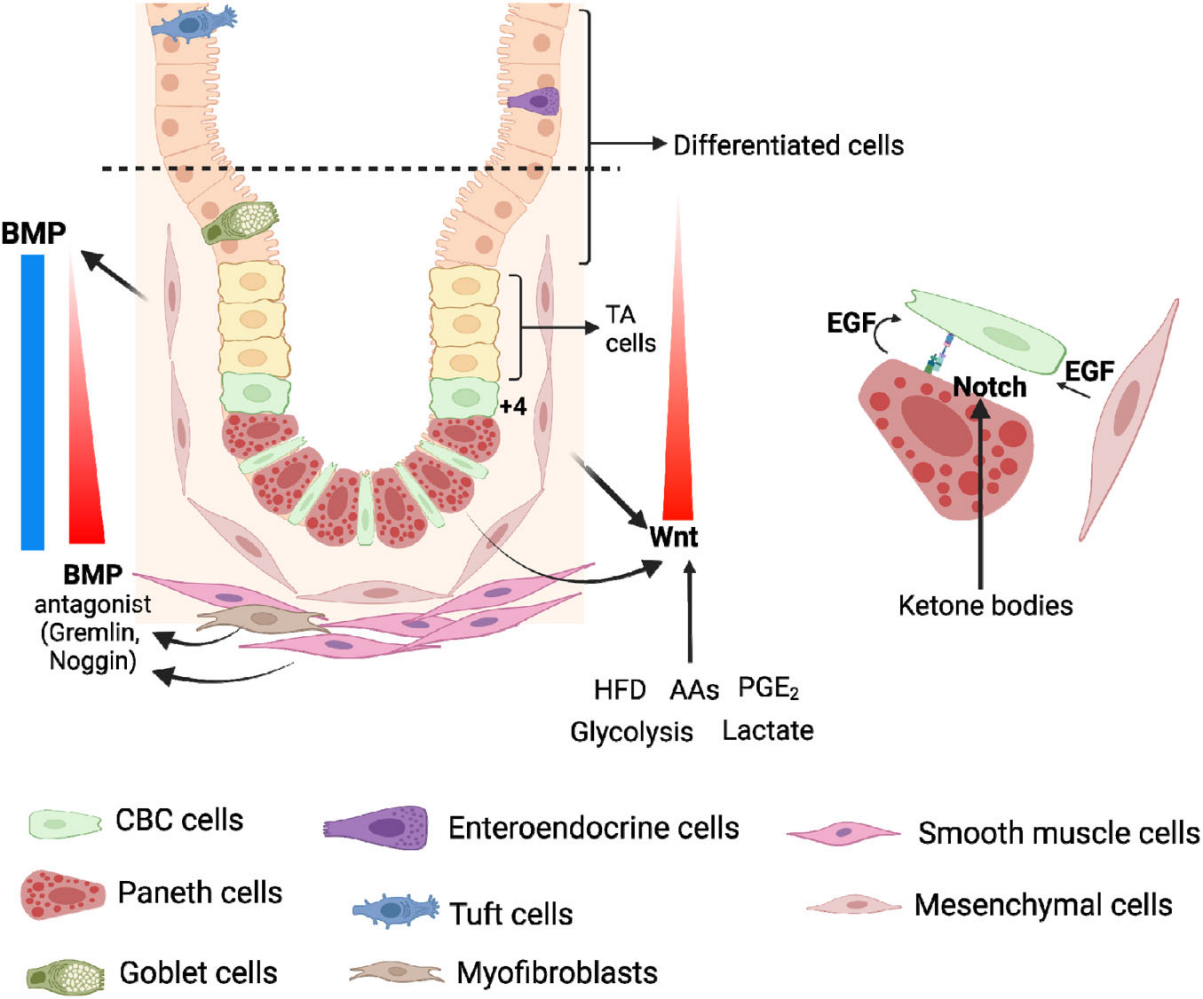
Major signalling pathways regulating intestinal stem cell (ISC) homeostasis. Wnt secreted by Paneth cells and mesenchymal cells enriches at the bottom of crypts and activates Wnt signalling in ISCs, thereby stimulating ISC proliferation. Bone morphogenetic protein (BMP) secreted by mesenchymal cells promotes ISC differentiation while suppressing ISC proliferation. The gradient of BMP activity is regulated by BMP antagonists produced by smooth muscle cells and myofibroblasts. Epidermal growth factor (EGF) secreted by Paneth cells and mesenchymal stromal cells supports ISC proliferation. Notch ligands secreted by Paneth cells facilitates the differentiation of ISCs into absorptive lineage. Several nutrients have been shown to activate some of these signalling pathways.

The proper maintenance of ISCs requires coordinated involvement of multiple signalling pathways, including WNT, bone morphogenetic protein (BMP), EGF, NOTCH and Yes‐associated protein (YAP) (Figure 1). The cooperation of these diverse pathways influences the fate of ISCs.

### 4.1 WNT

The WNT pathway is critical in ISC development and maintenance, exerting direct control over their proliferation and differentiation. The canonical WNT signalling pathway is governed by the transcriptional co‐activator β‐CATENIN, whose levels are regulated by a destruction complex comprising adenomatous polyposis coli (APC), AXIN, GSK3β and CK1α. In the absence of WNT ligands, the destruction complex phosphorylates cytoplasmic β‐CATENIN, marking it for ubiquitination‐mediated degradation. WNT ligands, such as WNT2B secreted from mesenchymal cells or WNT3 from Paneth cells,^28^, ^29^, ^30^ bind to the Frizzled‐LRP5‐LRP‐6 receptor complex, disrupting the destruction complex and facilitating the nuclear translocation of β‐CATENIN. Within the nucleus, β‐CATENIN binds to transcription factors of T cell factors/Lymphoid enhancer‐binding factor 1(TCF/LEF) family, and stimulates gene transcriptions.^31^ Experimental evidence has demonstrated that ablation of the key downstream transcription factor, TCF4, results in the complete loss of intestinal crypts.^32^ Similarly, overexpression of dickkopf WNT signalling pathway inhibitor 1 (DKK1) in adult mice leads to the total depletion of stem cells and decreased villus size and number.^33^ Conversely, transgenic expression of a potent WNT agonist, R‐SPONDIN1, which acts through the LGR4/5–WNT receptor complex, results in a dramatic hyperproliferation of intestinal crypts.^34^, ^35^ Moreover, simultaneous depletion of LGR4/5 leads to crypt disappearance.^16^ Thus, substantial evidence has underscored the pivotal role of the WNT pathway in developing and maintaining ISCs.

The distribution pattern of WNT ligands also contributes to the modulation of WNT signalling activity. Due to limited solubility, the concentrations of WNT ligands peak at the base of crypts and exhibit a gradient decrease along the crypt‐villus axis.^1^ Moreover, the quantity of surface‐anchored WNT3 ligands per cell reduces by half during cell division, adding another layer to the concentration gradient of WNT signalling.

### 4.2 BMP

The balance between ISC proliferation and differentiation cannot be achieved through the WNT pathway alone. Among the other pathways involved, BMP is known for its direct opposition to the WNT pathway.^36^ Unlike WNT, which stimulates ISC proliferation and suppresses differentiation, the BMP pathway promotes differentiation while restricting proliferation.^36^ Ablation of BMPR1A, a primary receptor for BMP2 and 4 in the intestine, results in uncontrolled stem cell proliferation and expansion.^36^ However, the exact underlying mechanism is still debatable. One study suggested that BMP can directly counteract WNT signalling through PTEN, effectively inhibiting β‐CATENIN and subsequent cell proliferation.^36^ However, recent evidence did not observe nuclear accumulation of β‐CATENIN after conditional *Bmpr1a* knockout, suggesting no direct inhibition of nuclear translocation of β‐CATENIN. Instead, BMP appears to influence stem cell progression through SMAD1/4 pathway.^37^

In the intestine, mesenchymal cells located in the intervillous/inter‐crypt region are the primary source of BMP, resulting in a seemingly uniform BMP distribution and suppression of stem cell proliferation throughout the crypt‐villus axis.^1^, ^38^, ^39^ However, multiple BMP antagonists secreted by myofibroblasts and smooth muscle cells, such as NOGGIN and GREMLIN, congregate at the base of the crypts. This effectively counters the suppressing effects of BMP on ISCs, creating a reversed gradient against WNT throughout the crypt‐villus axis.^29^, ^36^, ^40^

### 4.3 EGF

EGF plays a pivotal role in intestinal cell growth and proliferation through its receptor, EGFR, an ERBB‐type receptor tyrosine kinase.^1^ Upon binding, EGFR undergoes dimerization and autophosphorylation at the C‐terminal tyrosine residue, triggering a cascade of activation of several proliferation‐related pathways, including MAPK, PI3K, JNK and JAK.^41^ EGF, secreted by Paneth and stromal cells, is required for ex vivo intestinal organoid growth, suggesting the crucial roles of EGF in promoting ISC proliferation.^9^ It has been shown that blocking the EGF signalling pathway in organoid culture halts DNA replication in ISCs and converts them to a revertible quiescent state.^42^

### 4.4 NOTCH

The highly conserved NOTCH signalling functions as a switch for intestinal cell fate decisions.^43^, ^44^ NOTCH ligands, DLL1 and DLL4, secreted by Paneth cells, bind to their respective receptors through direct cell–cell contact, initiating the cleavage and release of NOTCH intracellular domain (NICD). Following its release, NICD translocates into the nucleus and relieves the inhibitory effect of transcription factor CSL, thereby activating NOTCH downstream target genes.^45^ NOTCH signalling affects cell lineage decisions by indirectly regulating MATH1 (also known as ATOH1), a transcription factor associated with secretory lineages. Activation of the NOTCH pathway stimulates the expression of HES1, thereby amplifying its inhibitory effect on MATH1. This ultimately directs progenitor cells towards the absorptive cell lineage. Conversely, suppressing NOTCH signalling decreases HES1 expression, activating MATH1 and steering more cells into the secretory lineage.^45^

### 4.5 YAP

YAP and transcriptional coactivator with PDZ‐binding motif (TAZ) are major downstream effectors of HIPPO pathway, which controls organ size by negatively regulating cell proliferation.^46^ Activation of HIPPO pathway stimulates the phosphorylation cascades and inactivates YAP/TAZ.^46^ Inactivated YAP/TAZ bind to 14‐3‐3 protein and are sequestered in the cytoplasm for degradation.^47^ Conversely, unphosphorylated YAP/TAZ translocate into the nucleus and interact with transcription factors from the TEAD family (TEAD 1–4), activating cell proliferation genes such as MYC and CTGF.^48^ YAP/TAZ are also reported to interact with Krüppel‐like factor 4 (KLF4) to promote the differentiation of stem and progenitor cells into goblet cells in the intestine.^49^

YAP/TAZ are essential for stem cell self‐renew and regeneration in various tissues. Under physiological conditions, YAP is predominantly expressed in crypt CBCs.^50^, ^51^ Mouse models with ubiquitous overexpression of a hyperactive YAP‐S127Amutant demonstrate activated NOTCH and WNT signalling and abnormal proliferation of undifferentiated cells in both crypts and villi,^52^ underscoring the growth‐promoting effect of YAP in ISCs. Surprisingly, Barry et al. showed that intestine‐specific activation of YAP suppresses ISC proliferation by constraining WNT signalling during intestinal regeneration, resulting in rapid crypt loss and Paneth cell mislocalisation.^53^ The seemingly contradictory effects of YAP activation in the intestine may be attributed to its potential paracrine signalling versus cell autonomous effects in ISCs.

YAP has also been shown to play a pivotal role in intestinal regeneration after injury. In dextran sulphate sodium (DSS)‐induced mouse colitis model and human CRC samples, YAP is activated through the GP130‐SFK (Src family kinase)‐YAP axis.^54^ Other studies utilising the same model and ex vivo organoid culture have identified FAK/SRC‐YAP activation as a major contributor to foetal‐like epithelium reprogramming during regeneration.^55^ Consistently, conditional loss of *Yap/Taz* or administration of inhibitors delays tissue repair, resulting in persistent ulcerations after DSS treatment.^55^ Another study has demonstrated that YAP is required for mobilising LGR5^−^ revival stem cells (revSCs) upon radiation (3 days post‐radiation) to promote de novo LGR5^+^ CBC production and intestine regeneration.^27^ To transition to a regenerative programme, YAP stimulates the EGFR pathway while inhibiting WNT signalling to suppress ISC markers and Paneth cell differentiation.^56^ In contrast, Barry et al. demonstrated that intestinal‐specific *Yap* depletion results in crypt hyperplasia and Paneth cell expansion in both small intestine and colon 7 days post‐irradiation.^53^ Barry et al. attributed this phenotype to a hyperactivated WNT network, as evidenced by the upregulation of several WNT target genes, such as *Cd44* and *Sox9*.^53^ These seemingly contradictory results suggest that YAP may play a crucial role in the early stages (~3 days post‐irradiation) of intestinal wound healing. During this period, WNT signalling will be temporarily suppressed to enable a transition to a regenerative/repair programme driven by EGFR. Subsequently, WNT signalling will resume activity ~7 days post‐irradiation to facilitate normal tissue proliferation. Taken together, these findings underscore the critical role of YAP/TAZ in maintaining intestinal homeostasis and driving tissue regeneration after injury.

It has been well established that these signalling pathways are involved in both maintaining intestinal homeostasis and facilitating regeneration after injury. However, their activities are differentially regulated between homeostatic and regenerative states. Unlike homeostasis, which strives for a proliferation‐differentiation balance, early‐stage regeneration requires disrupting this balance in favour of proliferation. As a result, pathways like YAP, EGF and NOTCH are activated, while others (such as BMP) are attenuated. Conversely, as regeneration progresses, the pro‐proliferation pathways recede, and pro‐differentiation pathways will be elevated.

## 5 METABOLIC CONTROL OF ISC HOMEOSTASIS

Accumulating evidence has demonstrated that the fate of ISCs is dynamically influenced by both internal metabolic pathways and external nutritional signals, which encompass various factors, including macronutrients (lipids and carbohydrates), micronutrients (such as vitamins) and metabolites derived from gut microbiota. In the following section, we provide an overview of recent advances in understanding how metabolism regulates the balance of ISC proliferation and differentiation.

### 5.1 dietary regulation of ISC function

#### 5.1.1 Fasting, fast‐refeeding and calorie restriction

**FIGURE 2:**
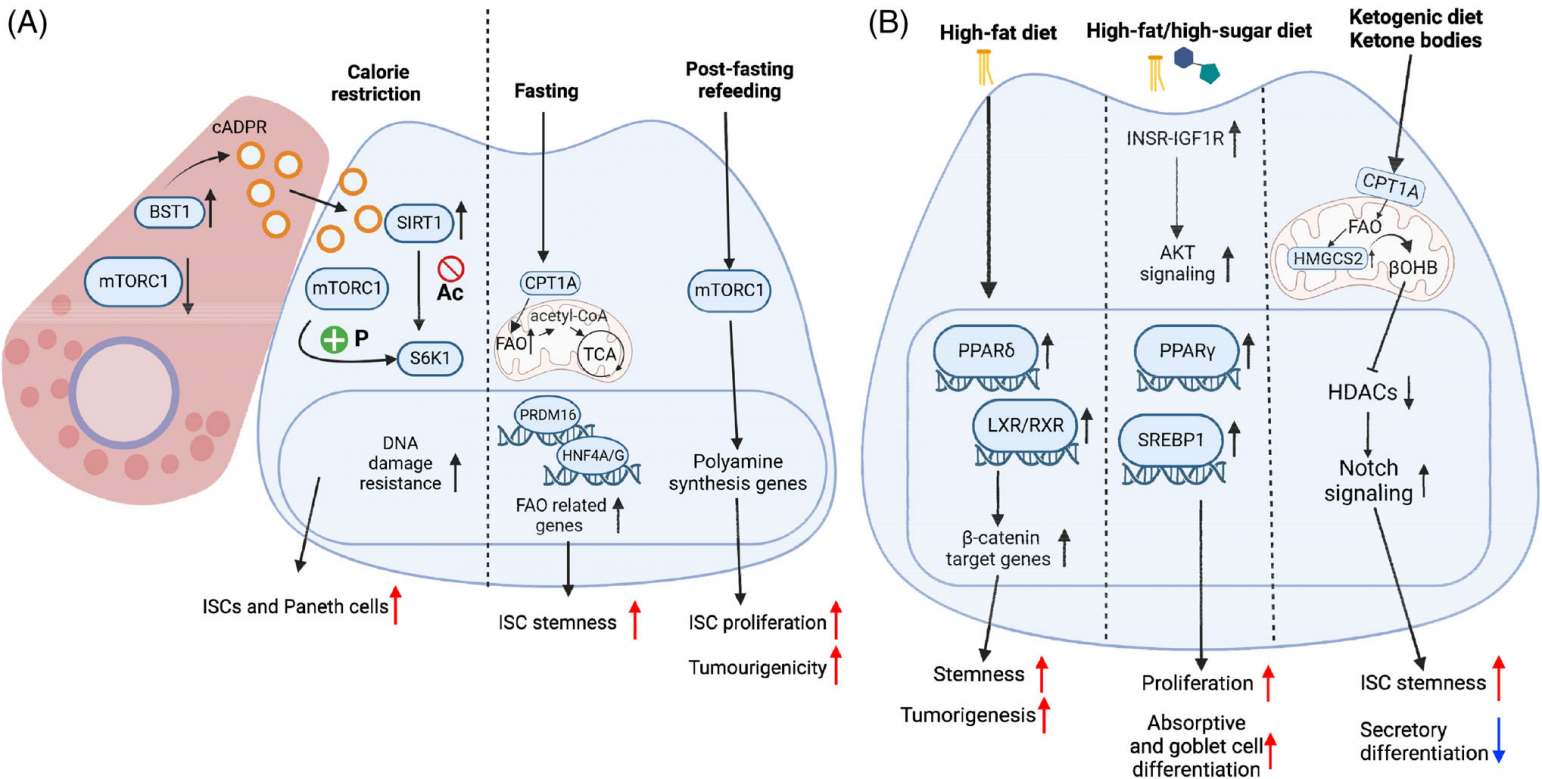
Dietary patterns regulate intestinal stem cell (ISC) function. (A) Fasting, post‐fast refeeding and calorie restriction. Fasting enhances ISC functions by initiating fatty acid oxidation (FAO) programme, dependent on carnitine palmitoyltransferase 1A (CPT1A). Other regulators such as PRDM16 and HNF4A/G also regulate ISCs by modulating FAO. Post‐fast refeeding stimulates mTORC1 and activates protein synthesis through a polyamine metabolism programme. As a result, both ISC proliferation and tumorigenesis are elevated. During calorie restriction (CR), Paneth cell secrets paracrine factor cyclic ADP ribose (cADPR) due to the reduced function of mechanistic target of rapamycin complex 1 (mTORC1). cADPR enters ISCs and promotes the proliferation of both ISCs and Paneth cells through SIRT/mTORC1‐S6K1 signalling. CR also enhances DNA damage resistance in reserve ISCs, preserving regenerative capacities. (B) High‐fat, high‐fat/high‐sugar and ketogenic diet. High‐fat diet (HFD) promotes ISC proliferation by activating β‐catenin target genes through peroxisome proliferator–activated receptor δ (PPARδ) and LXR/FXR signalling. Furthermore, PPARδ enables progenitor cells to regain stem cell characteristics, promoting tumorigenesis. High‐fat/high‐sugar diet (HFHSD) induces mucosal changes and intestinal maladaptation by activating sterol regulatory element‐binding protein 1 (SREBP1, for fatty acid synthesis), PPARγ signalling, and insulin receptor‐Igfr‐Akt pathway. Ketone bodies derived from ketogenic diets (KTD) or fasting affect ISC stemness and differentiation through 3‐hydroxy‐3‐methylglutaryl‐ CoA synthetase 2 (HMGCS2)‐class 1 histone deacetylase (HDAC)‐Notch signalling.

It is well established that ISC homeostasis is modulated by nutrient availability. In the intestine, 24 h fasting enhances ISC function^57^ (Figure 2A). Gene expression analysis revealed that fasting triggers fatty acid oxidation (FAO) programmes in ISC, involving the rate‐limiting enzyme, carnitine palmitoyltransferase 1A (CPT1A). Disruption of *Cpt1a* not only restricts ISC‐enhancing effects from fasting but also decreases ISC numbers and function over time. However, the role of FAO in the intestine is not solely attributed to CPT1A. Recently, Stine et al. have identified a transcription factor, PRDM16, which directly regulates FAO in the crypts of the upper intestine.^58^ Acute deletion of *Prdm16* in mice compromises epithelial differentiation, activates progenitor apoptosis, and impairs intestinal cell development and maintenance. Other regulators of FAO have also been reported to regulate ISC homeostasis. Transcription factors, hepatocyte nuclear factor 4α (HNF4A) and HNF4G, directly activate genes involved in FAO and are required for ISC renewal.^59^ Deletion of both *Hnf4a* and *Hnf4g* in the intestine leads to the loss of LGR5^+^ ISCs. Interestingly, post‐fast refeeding has also been shown to enhance ISC and progenitor cell function **(**Figure 2A **)**. Refeeding for 24 − 72 h after 24 h‐fasting shows augmentation in both ISC and progenitor cell proliferation.^60^ Further investigations showed that fast‐refeeding activates protein synthesis via a mammalian target of rapamycin complex 1 (mTORC1)‐stimulated polyamine metabolism programme. Protein synthesis also increases the risk of tumorigenesis. Indeed, post‐fast refeeding in *Apc*‐null mice enhances polyp formation in both small and large intestines.^60^

On the other hand, prolonged calorie restriction (CR) not only augments ISC function but also increases OLFM4^+^ stem cells and Paneth cell numbers.^57^, ^61^ CR exerts its effects mainly through mTORC1 (Figure 2A). Yilmaz et al. showed that CR enhances ISC function by reducing mTORC1 signalling in Paneth cells, but not in ISCs.^61^ During CR, mTORC1 signalling is suppressed in Paneth cells, stimulating the production of bone stromal antigen 1 (BST1), an enzyme that produces cyclic ADP ribose (cADPR), a paracrine factor secreted by Paneth cells. cADPR activates SIRT1 in ISCs, which deacetylates S6K1 and facilitates its phosphorylation by mTORC1. Phosphorylated S6K1 induces protein synthesis and promotes ISC proliferation.^62^, ^63^ Interestingly, mTORC1 has also been shown to regulate stem cell proliferation and govern the sensitivity of reserve ISCs to DNA damage.^63^ Studies by Yousefi et al. suggested that CR actively inhibits mTORC1 in reserve ISCs, enhancing their resistance to DNA damage and thereby preserving their regenerative capacities.^63^

#### 5.1.2 Ketogenic diet and ketone bodies

During extended period of fasting, the body employs ketogenesis to maintain energy levels through the breakdown of fatty acids and ketogenic amino acids, such as Leucine and Lysine.^64^ Notably, ketone bodies can also function as signalling molecules and influence stem cell maintenance. A recent study has highlighted the role of ketogenic diet and beta‐hydroxybutyrate (βOHB) in regulating ISC stemness by modulating NOTCH signalling pathway^65^ (Figure 2B). Mice on a ketogenic diet have elevated crypt βOHB levels and ISC numbers. The rate‐limiting enzyme in βOHB synthesis, 3‐hydroxy‐3‐methylglutaryl‐CoA synthetase 2 (HMGCS2), is highly expressed in LGR5^+^ cells rather than differentiated cells. The elevated expression of HMGCS2 by ketogenic diet feeding results in higher βOHB levels. *Hmgcs2* deletion impairs ISC self‐renewal, reduces ISC numbers and drives differentiation towards secretory cell lineage, suggesting that βOHB is crucial for maintaining a balance between stem cell self‐renewal and differentiation. Further study revealed that βOHB inhibits class 1 histone deacetylase to enhance NOTCH signalling, thereby guiding ISC self‐renewal and differentiation **(**Figure 2B **)**.

#### 5.1.3 High‐fat diet

While numerous studies have documented the link between colon cancer and obesity, the mechanisms by which ISCs and progenitors are altered to promote dysplasia formation remain elusive. In a noteworthy study by Yilmaz et al., mice challenged with long‐term high‐fat diet (HFD) feeding exhibit augmented ISC proliferation and reduced reliance on Paneth cells for organoid formation,^66^ providing a novel perspective on how obesity may contribute to the development of colon cancer. Transcriptomic sequencing revealed the enrichment of motifs of nuclear receptors, peroxisome proliferator‐activated receptor δ (PPARδ), and liver/retinoid X receptor (LXR/RXR). The elevated binding of PPARδ and LXR/RXR to their respective targets activates common ISC proliferation pathways, such as WNT/β‐CATENIN pathway (Figure 2B). Moreover, PPARδ also contributes to progenitor cells acquiring stem cell traits, thereby promoting tumour initiation. In another study, Aliluev et al. demonstrated that an obesogenic high‐fat/high‐sugar diet (HFHSD) induces hyperproliferation of ISCs and progenitor cells, promotes their differentiation into absorptive and goblet cell lineage, which contributes to the pathogenesis of the diet‐induced obesity and metabolic syndrome.^67^ Mechanistically, HFHSD increases fatty acid synthesis, activates PPAR and Insulin receptor‐IGFR‐AKT pathway, which leads to mucosal changes and intestinal maladaptation (Figure 2B). Although these studies have demonstrated that diets significantly impact ISC function, it remains to be explored how specific components within these diets affect ISCs.

### 5.2 Lipid metabolism in ISC regulation

#### 5.2.1 Fatty acid synthesis

Recent research has unveiled the significance of fatty acid synthesis in impacting ISC maintenance.^68^ Intestinal‐specific deletion of Acetyl‐CoA carboxylase1 (ACC1), a rate‐limiting enzyme in de novo fatty acid synthesis, has been shown to lead to the loss of crypt structures and a selective reduction in LGR5^+^ stem cells.^68^ Mechanistically, ACC1 deletion hampers the activation of the PPARδ/β‐CATENIN signalling pathway. Interestingly, supplementation with fatty acids is sufficient to rescue the deficiency in organoid formation in *Acc1*‐deficient crypts, suggesting that ISC function is dependent on the availability of lipids. Moreover, inhibition of ACC1 also limits tumour formation in a DSS/AOM‐induced inflammatory colon cancer model. These studies highlight the critical role of lipogenesis in maintaining ISC function and provide a potential approach for colon cancer therapy.

#### 5.2.2 Membrane phospholipid remodelling

**FIGURE 3:**
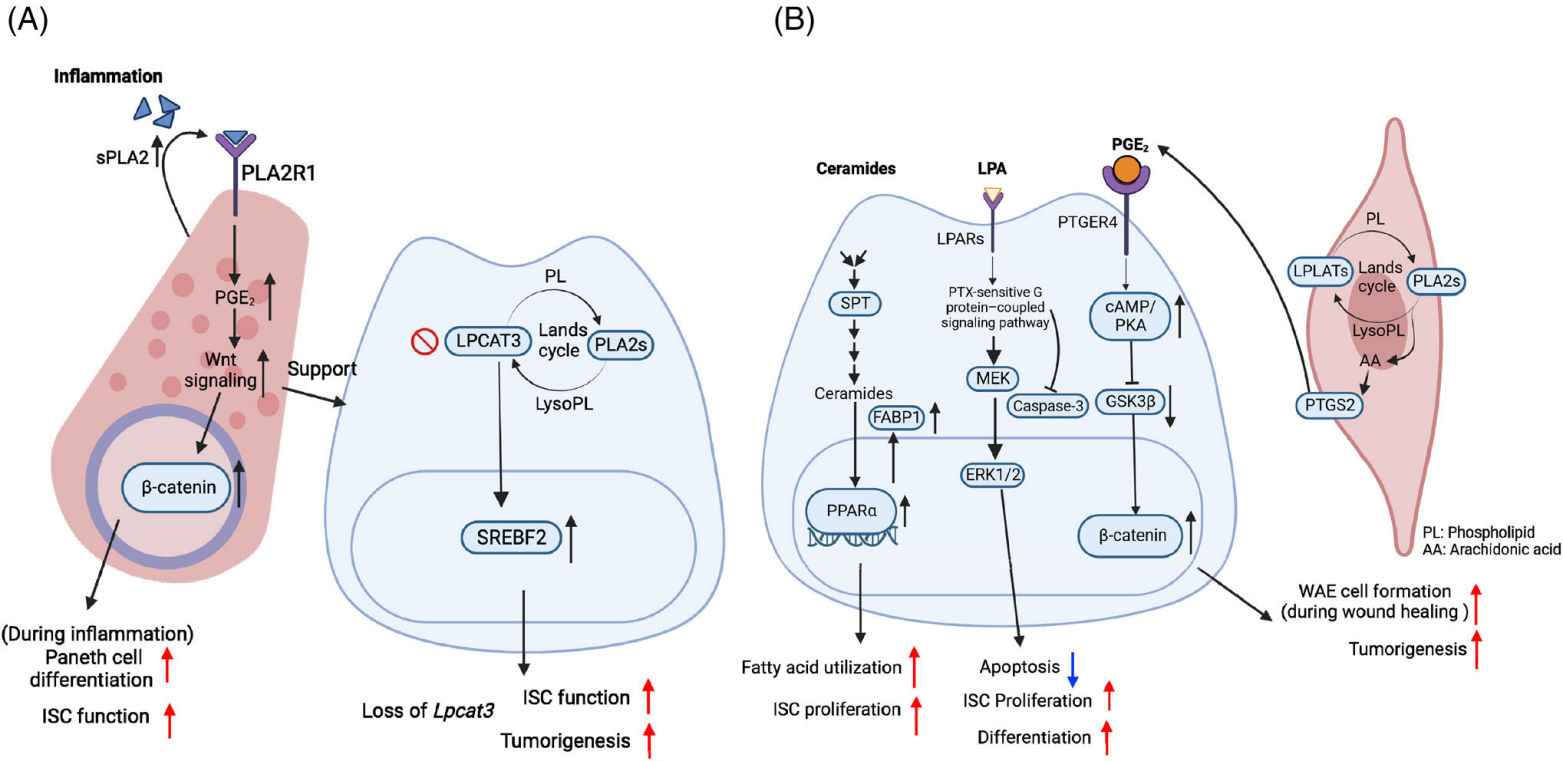
Lipid metabolism in intestinal stem cell (ISC) regulation. (A) Phospholipids (PL) remodelling. Lysophosphatidylcholine acyltransferase 3 (LPCAT3) regulates ISC function and tumorigenesis by modulating membrane phospholipid composition and sterol regulatory element‐binding protein 2 (SREBP2) activity. Loss of *Lpcat3* increases membrane saturation and SREBP2‐mediated cholesterol biosynthesis, thereby enhancing ISC stemness and tumorigenesis. Phospholipases A2 (PLA2) indirectly enhances ISC functions by modulating Paneth cell Wnt signalling during inflammation. Under homeostasis, intracellular PLA2 suppresses Paneth cell Wnt signalling through activation of YAP, thereby indirectly inhibiting Paneth cell differentiation and ISC proliferation. Upon inflammation, PLA2 is secreted autocrinally and acts on Paneth cell receptor PLA2R1 to promote prostaglandin E2 (PGE_2_) production and Wnt signalling, thereby enhancing Paneth cell maturation and its niche function for ISC proliferation and tumorigenesis. (B) Lipid signalling molecules. Ceramides are essential for ISC homeostasis. Disrupting ceramide biosynthetic enzyme serine palmitoyl transferase (SPT) inhibits ISC proliferation. Ceramides enhance fatty acid utilisation and ISC proliferation by activating PPARα. Lysophosphatidic acid (LPA) protects ISCs from radiation‐ and chemotherapy‐induced apoptosis through caspase‐3/CPP32 inhibition. Exogenous LPA enhances both ISC proliferation and differentiation through Ras–MEK‐ERK1/2 pathway. PGE_2_ regulates intestinal repair during wound healing. PGE_2_, produced by mesenchymal PTGS2, promotes wound‐associated epithelial (WAE) cell differentiation by activating Wnt signalling through PTGER4‐cAMP/PKA‐ β‐catenin axis.

Phospholipids (PLs), composed of two hydrophobic fatty acids and one hydrophilic head group, are major components of biological membranes that provide platforms for cellular processes.^69^ The composition of PLs encompasses a wide array of species depending on the length and degree of saturation of fatty acyl chains, which determine the biophysical properties of membranes and thereby influence cell functions.^70^ A dynamic remodelling process referred to as Lands' cycle controls PL composition. In this cycle, PLs will be hydrolysed by phospholipase A2 (PLA2) and re‐acylated by lysophospholipid acyltransferases with different fatty acids.^71^ Lysophosphatidylcholine acyltransferase 3 (LPCAT3), the most abundant LPCAT in the intestine, is the key enzyme catalysing the incorporation of polyunsaturated fatty acids to the sn‐2 site of lysophosphatidylcholine to produce polyunsaturated phosphatidylcholine.^72^ It has been shown that membrane PL metabolism in the intestine modulates ISC function and tumorigenesis^73^, ^74^, ^75^ (Figure 3A). Acute knockout of *Lpcat3* in mouse intestines leads to crypt hyperproliferation.^75^ Mechanistically, loss of *Lpcat3* stimulates post‐transcriptional activation of sterol regulatory element‐binding protein 2 (SREBP2) and enhances cholesterol biosynthesis. Inhibition of cholesterol biosynthesis normalises crypt hyperproliferation in *Lpcat3* KO mice. Moreover, *Lpcat3* depletion and increasing cellular cholesterol levels dramatically promote tumour growth in *Apc* ^*Min/+*^ mice, providing a potential link between dietary lipids and tumorigenesis in the gut.

Interestingly, phospholipases A2 (PLA2), another enzyme involved in PL remodelling, has also been shown to modulate ISC function^76^ (Figure 3A). However, unlike LPCAT3 which regulates ISC homeostasis by remodelling membrane PL composition, PLA2 functions as an autocrine niche factor secreted from Paneth cells. Under homeostasis, a major portion of PLA2 remains intracellular, suppressing WNT signalling through YAP activation in Paneth cell, thereby indirectly inhibiting Paneth cell differentiation and ISC proliferation. In contrast, in the context of inflammation, PLA2 is secreted into intestinal lumen in an autocrine manner. This promotes Paneth cell prostaglandin E2 (PGE_2_) production and WNT signalling, augmenting Paneth cell maturation, ISC proliferation and tumorigenesis.

#### 5.2.3 Lipid signalling molecules

In the gut, lipid signalling molecules, including ceramides, lysophosphatidic acid (LPA) and prostaglandin E2 (PGE_2_), have been shown to play profound roles in ISC proliferation (Figure 3B).

Ceramide is a major sphingolipid species that gives rise to all complex sphingolipids. The de novo synthesis of ceramide involves serine palmitoyl transferase (SPT). Deletion of SPT subunit, SPTLC2, inhibits ISC proliferation in mice.^77^ Similarly, disruption of SPT homologue *lace* or the ceramide synthase homologue *schlank* in *Drosophila* recapitulates the phenotype.^77^ Conversely, treating *Sptlc2*‐knockout organoids with a ceramide analogue, C_2_‐ceramide, rescues ISC proliferation. RNA‐seq analysis revealed that ceramide activates fatty acid‐binding protein 1 (FABP1) through PPARα, which enhances fatty acid utilisation and ISC proliferation.^77^

LPA is a phospholipid signalling molecule that promotes cell proliferation through LPA receptors. An earlier study has established the protective role of LPA against radiation‐ and chemotherapy‐induced apoptosis in enterocyte cell lines and mouse models.^78^ Mechanically, LPA triggers tyrosine phosphorylation of the EGFR, activates a pertussis toxin‐sensitive G protein–coupled signalling pathway, and inhibits the caspase‐3/CPP32 activation.^78^ Furthermore, a recent study found that LPA treatment enhances RAS–MEK–ERK‐induced cell proliferation and differentiation in intestinal organoids.^79^ Similarly, deletion of *Lpar5*, a major intestinal LPA receptor hampers ISC proliferation and promotes crypt apoptosis.^80^ These studies strongly demonstrated the promoting effect of LPA on ISC proliferation, both under homeostasis and in response to injury.

PGE_2_ is an unstable lipid signalling molecule converted from arachidonic acid by prostaglandin‐endoperoxide synthase 2 (PTGS2).^81^ PGE_2_ is produced by a mesenchymal pericryptal fibroblasts, and has been shown to regulate WNT signalling pathway through cAMP/PKA.^82^ During wound healing in the intestine, PGE_2_ signalling through one of its receptors, PTGER4, promotes the differentiation of ISCs into wound‐associated epithelial (WAE) cells and facilitates wound repair.^83^ In contrast, inhibition or absence of PGE_2_‐PTGER4 signalling leads to the differentiation of ISCs into enterocytes, suggesting that PGE_2_ regulates ISC cell fate determination. PGE_2_ has also been shown to be involved in tumour development in the gut. In the process of tumorigenesis, hydrophobic PGE_2_ diffuses across the basal lamina matrix and binds to tumour‐initiating stem cells (SCA‐1^+^ cells) via PTGER4.^81^ Activation of PTGER4 inhibits HIPPO pathway, resulting in the dephosphorylation and activation of YAP, thereby promoting stemness and tumorigenesis. Interestingly, ablation of PTGS2 in fibroblasts is sufficient to prevent tumorigenesis. Similarly, deletion of *Ptger4* in epithelial cells disrupts the regenerative reprogramming of ISCs and prevents SCA‐1^+^ cell proliferation and tumour initiation in *Apc* ^*Min/+*^ mice.^81^

#### 5.2.4 Cholesterol and its derivatives

As described above, increasing cellular cholesterol levels through SREBP2 activation or excess dietary cholesterol promotes ISC proliferation and self‐renewal,^75^ suggesting that cholesterol acts as a mitogen for ISCs. Conversely, inhibition of cholesterol biosynthesis dramatically impairs crypt growth. Intestinal ablation of SREBP cleavage‐activating protein (SCAP), an ER protein required for cleavage and activation of SREBPs, blocks SREBP activation and lipid biosynthesis, resulting in severe crypt growth failure and enteropathy in mice.^84^

**FIGURE 4:**
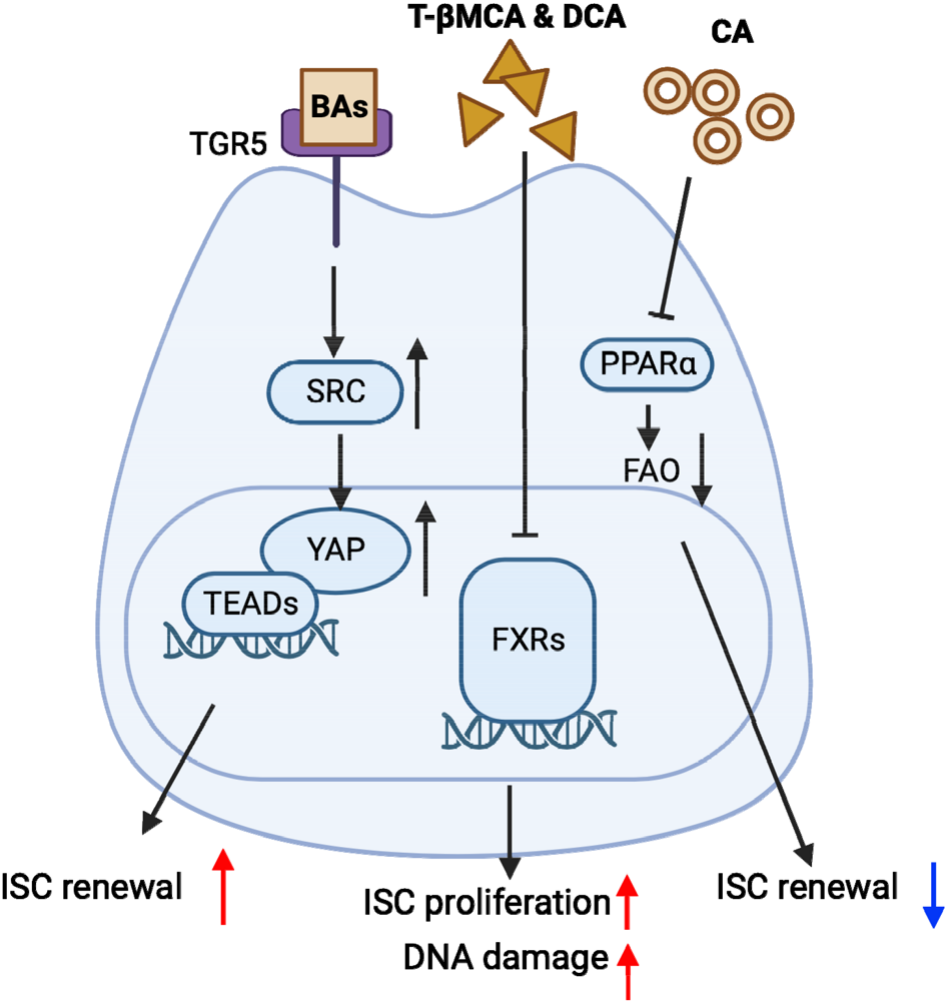
Bile acids (BAs) in intestinal stem cell (ISC) regulation. During intestinal injury, BAs promote ISC regeneration by binding to the G protein‐coupled bile acid receptor 1 (GPBAR1, also known as TGR5), and activating the proliferative YAP signalling. Tauro‐β‐muricholic acid (T‐βMCA) and deoxycholic acid (DCA) promote colorectal cancer progression by suppressing farnesoid X receptor (FXR) and promoting ISC proliferation and DNA damage. Cholic acid (CA) suppresses ISC renewal by deactivating peroxisome proliferator‐activated receptor α (PPARα) and fatty acid oxidation.

Metabolites derived from cholesterol, such as bile acids (BAs), have also been found to play an important role in maintaining ISC homeostasis (Figure 4). Schoonjans et al. demonstrated that BAs stimulate ISC regeneration in response to injury by activating the G protein‐coupled bile acid receptor 1 (GPBAR1, also called TGR5).^85^ Activation of TGR5 subsequently triggers SRC/YAP pathway to promote ISC renewal and drive regeneration. Interestingly, Fu et al. reported that tauro‐β‐muricholic acid and deoxycholic acid antagonise intestinal farnesoid X receptor (FXR) function, induce proliferation and DNA damage in LGR5^+^ cells, and thereby promote colorectal cancer progression.^86^ Conversely, selective activation of FXR in the intestine suppresses abnormal LGR5^+^ cell growth and impedes CRC progression. Another study by Chen et al. revealed that the accumulation of cholic acid in the human and mouse guts suppresses ISC renewal.^87^ Mechanistically, cholic acid inhibits FAO by deactivating PPARα.^87^ On the other hand, suppression of hepatic Cytochrome P450 8B1 (CYP8B1), an enzyme synthesising cholic acid, restores ISC renewal and alleviates colitis in mice.^87^ These findings indicate that different BAs can have opposite effects on ISC function depending on the conditions.

### 5.3 Carbohydrate metabolism in ISC regulation

**FIGURE 5:**
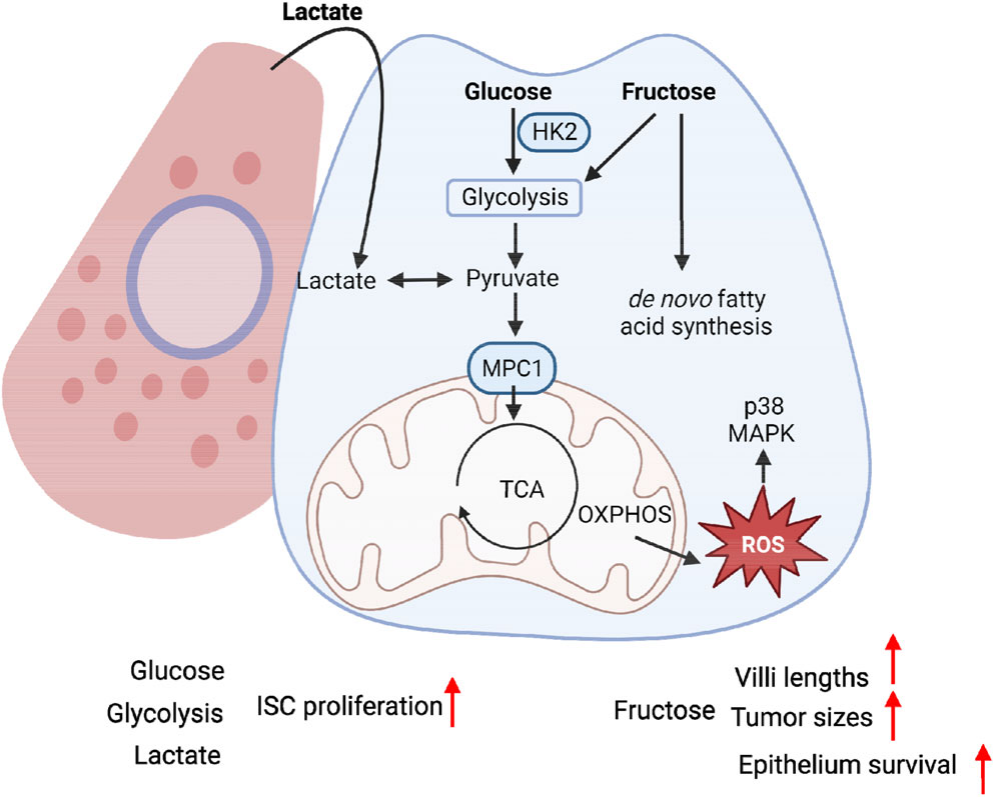
Carbohydrate metabolism in intestinal stem cell (ISC) regulation. Glucose promotes ISC proliferation through enhancing glycolysis, TCA cycle and oxidative phosphorylation (OXPHOS). Glycolysis in Paneth cells produces lactate to support higher OXPHOS rates in ISCs, thereby promoting their proliferation. Mechanistically, OXPHOS helps maintain a balance between ISC proliferation and differentiation by reactive oxygen species‐mediated activation of p38 MAPK pathway. Fructose promotes villi elongation and cell survival by enhancing glycolysis and de novo fatty acid synthesis.

It is well established that stem cells exhibit higher glycolysis and lower oxidative phosphorylation (OXPHOS) compared to their differentiated progeny.^88^, ^89^ Studies have shown that ISC proliferation is enhanced by glucose but mitigated by glycolytic pathway inhibitor 2‐deoxy‐d‐glucose^90^ (Figure 5). Similarly, knockout of hexokinase‐2 (*Hk2*), a target gene of WNT signalling pathway encoding the first enzyme in glycolytic pathway, significantly reduces glycolysis and lactate production in intestinal organoids, resulting in attenuated ISC self‐renewal.^89^

Interestingly, despite their preference for glycolysis, ISCs display elevated mitochondria activity compared to Paneth cells, as evidenced by an increase in pyruvate/lactate ratio.^91^, ^92^ On the other hand, Paneth cells support stem cell function by providing lactate to sustain the higher mitochondrial OXPHOS in ISCs (Figure 5). Consequently, inhibition of mitochondrial OXPHOS in LGR5^+^ ISCs or suppressing glycolysis in Paneth cells impairs crypt organoid formation.^91^ Mechanistically, OXPHOS stimulates p38 MAPK pathway by generating reactive oxygen species (ROS), which promotes ISC differentiation and organoid formation, thereby maintaining a balance between the proliferation and differentiation of ISCs. These findings suggest that cellular redox state modulates ISC proliferation and differentiation, emphasising the importance of maintaining a specific level of ROS for ISC homeostasis. The oxidation of carbohydrates involves the import of pyruvate into mitochondria via mitochondrial pyruvate carrier (MPC). Deleting MPC1 in mice increases stem cell proliferation and ISC population.^92^ Conversely, overexpression of MPC1 effectively suppresses ISC proliferation (Figure 5), further reinforcing the involvement of pyruvate metabolism in regulating ISC homeostasis.

Fructose constitutes a significant component in the western diet, and studies have revealed its association with an increased intestinal tumour burden and growth in various mouse models.^93^, ^94^ Mice fed on high fructose corn syrup (HFCS) show elongated villi and increased tumour sizes.^93^, ^94^ Interestingly, these effects are not due to increased cell proliferation, but rather an augmentation in cell survival, particularly in hypoxic tissues like tumours.^94^ On the other hand, Goncalves et al. suggested that HFCS promotes glycolysis and de novo fatty acid synthesis, thereby supporting tumour growth.^93^ Taken together, these data underscore the importance of glycolysis and mitochondrial OXPHOS in modulating ISC function (Figure 5).

### 5.4 Amino acids metabolism in ISC regulation

Amino acids (AAs) are essential building blocks for rapidly expanding tissues, including intestinal epithelium, that require massive protein synthesis. AAs such as glutamine (Gln), glutamate (Glu), Arginine (Arg), methionine (Met) and valine (Val) are essential in ISC proliferation.^95^, ^96^, ^97^, ^98^, ^99^, ^100^, ^101^ Dietary supplementation of alanyl‐glutamine (Ala‐Gln) has been shown to rescue crypt depletion in malnourished mouse pups.^98^ Treating organoids with Gln or Ala‐Gln enhances organoid expansion through an mTOR‐dependent pathway.^98^ Similarly, Gln supplementation ameliorates early weaning‐induced epithelial atrophy by enhancing WNT/β‐CATENIN signalling in ISCs.^100^ Additionally, Glu is reported to boost ISC and ex vivo organoid formation in *Drosophila*.^95^

l‐arginine (Arg) is an essential AA for enterocyte protein synthesis. Studies using mouse ex vivo organoids revealed that Arg deficiency diminishes ISC and Paneth cell populations.^97^ Interestingly, Arg treatment promotes ISC proliferation in vivo, but has no effects on isolated LGR5^+^ ISC population and their organoid‐forming capabilities, indicating a non‐cell‐autonomous effect.^96^ Further studies showed that Arg supplementation augments the secretion of WNT3A and WNT2B from Paneth cells and CD90^+^ stromal cells, respectively, to promote ISC proliferation.^96^, ^97^ Furthermore, Arg supplementation protects mouse intestinal epithelia from 5‐Fluorouracil‐ and TNFα‐induced damage in vivo and ex vivo.^96^, ^97^ Therefore, Arg supplementation could be a therapeutic approach for intestinal injury caused by chemotherapy.

Other essential AAs, Met and Val, have also been demonstrated to impact ISC proliferation.^99^ Ex vivo organoid cultures depleted of Met or Val restrict proliferating ISC numbers.^99^ Moreover, Met depletion reduces *Lgr5* RNA expression and promotes the differentiation of secretory cell lineages, such as goblet cells and Paneth cells.^99^

### 5.5 Vitamins in ISC regulation

Recent studies have elucidated the impact of the active form of vitamin D, vitamin D_3_, on the maintenance of ISCs and inflammation in crypts. Notably, the vitamin D receptor (VDR) is prominently expressed in LGR5^+^ CBCs.^102^ Mice fed an NWD1, a pro‐tumorigenic diet with higher fat and lower vitamin D_3_ and calcium, show impaired LGR5^+^ ISC function as evidenced by decreased proliferation and differentiation capacity.^103^ Intriguingly, introducing vitamin D3 and calcium supplementation to the diet abrogates the adverse impact of NWD1 on ISC function. Similarly, another study has demonstrated that vitamin D3 enhances the differentiation of ISCs into various specialised epithelial cells, including enterocytes, Paneth, goblet and enteroendocrine cells.^104^ Concurrently, vitamin D3 treatment suppresses ISC stemness due to increased ER stress.

*Vdr* knockout mouse models have further validated the role of vitamin D in ISC regulation. Consistent with the effect of vitamin D3 deficiency on ISC function, Peregrina et al. showed that the inactivation of VDR in LGR5^+^ ISCs obstructs their differentiation.^103^ Vitamin D3/VDR signalling pathway also protects ISCs and progenitor cells against DNA damage and apoptosis induced by ionising radiation. Notably, *Vdr* deficiency activates the PMAIP1‐mediated apoptotic pathway within ISCs and progenitor cells following ionising radiation‐induced damage.^105^ These studies indicate that vitamin D/VDR likely plays a pivotal role in modulating stem cell function.

While vitamin A (retinol) and its metabolites (4‐OH‐RA and 4‐Oxo‐RA) are recognised for their critical roles in cell development processes such as haematopoietic stem cell maintenance and intestinal dendric cell antigen presentation,^106^, ^107^, ^108^ limited research has linked vitamin A with ISC homeostasis. A study conducted in weaned piglets revealed that dietary vitamin A significantly increases villi height and crypt depth.^109^ Ex vivo organoid assay indicated that vitamin A treatment significantly reduces organoid budding rates, suggesting that vitamin A may affect ISC differentiation and stemness.^109^ However, the underlying mechanisms remain unclear.

### 5.6 gut microbiota‐derived metabolites in ISC regulation

**FIGURE 6:**
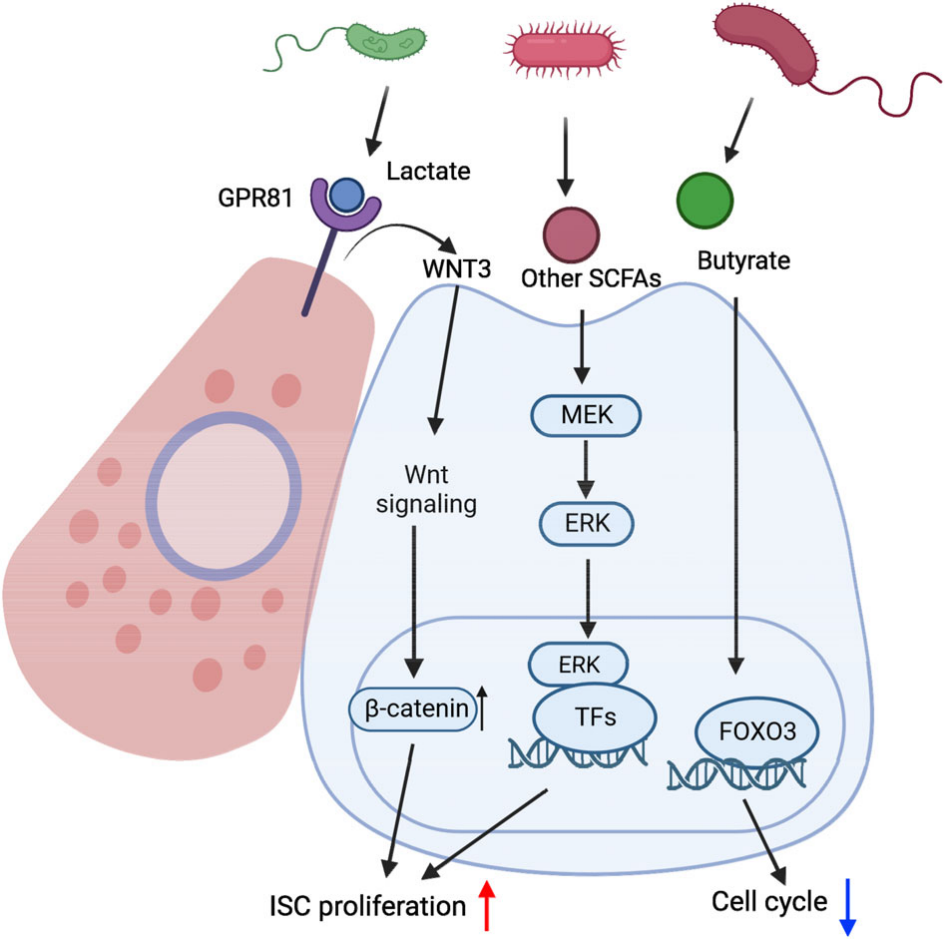
Gut microbiota‐derived metabolites in intestinal stem cell (ISC) regulation. Butyrate negatively regulates cell cycle by activating Forkhead box O3 (FOXO3). Other short chain fatty acids (SCFAs) enhance ISC proliferation by promoting MEK–ERK pathway. Lactate produced by lactic acid‐producing bacteria (LAB) promotes ISC proliferation by interacting with G‐protein‐coupled receptor 81 (GPR81) located on Paneth cells that secrete WNT3. WNT3, in turn, activates Wnt/β‐catenin signalling in adjacent ISCs, triggering their proliferation.

Growing evidence has demonstrated that gut microbiota plays a crucial role in modulating host physiology and contributing to the pathogenesis of various diseases.^110^ As described above, secondary BAs, such as deoxycholic acid, modulate ISC function through its receptor TGR5.^85^ Other metabolites produced by gut bacteria, including short‐chain fatty acids (SCFAs) and lactate, have also been reported to regulate ISC proliferation and differentiation^111^, ^112^, ^113^ (Figure 6). One of the SCFAs, butyrate, is a potent inhibitor of stem/progenitor cell proliferation in the colon.^111^, ^113^ Butyrate potentiates the activity of transcription factor forkhead box O3 (FOXO3), which drives the expression of negative cell‐cycle regulators.^111^ Interestingly, SCFAs have been shown to have the opposite effect on stem/progenitor cell proliferation in small intestine.^113^, ^114^, ^115^ Park et al. showed that the proliferation and migration of intestinal epithelial cells in the crypts are attenuated in germ‐free mice. At the same time, oral administration of SCFAs restores the turnover of intestinal epithelial cells in these mice and promotes organoid growth in vitro.^114^ Mechanistically, SCFAs enhance MEK–ERK signalling pathway to promote ISC proliferation. Therefore, SCFAs, especially butyrate, may exert cell‐type‐specific effects in different regions of the intestine.

In another study, Lee et al. revealed the role of lactic acid‐producing bacteria (LAB), such as *Bifidobacterium* and *Lactobacillus* spp., in supporting ISC‐mediated epithelial regeneration in the intestine.^116^ LAB‐generated lactate effectively reaches the bottom of the crypt base, engaging with GPR81 on Paneth and stromal cells. The activation of GPR81 in Paneth and stromal cells triggers the secretion of WNT3, which activates WNT/β‐CATENIN signalling in neighbouring ISCs and thereby promotes ISC proliferation. As the interest in the faecal microbiota as a therapeutic approach grows, further studies are necessary to enhance our understanding of how gut microbiota and their metabolites influence gut physiology and function.

## 6 CONCLUSIONS

The regulation of adult ISCs involves an intricate feedback loop incorporating intrinsic signalling pathways like WNT, EGF and BMP, alongside extrinsic factors such as various metabolites, including lipids, carbohydrates, and microbe‐derived metabolites. The dynamic interplay between internal pathways and external influences is essential for maintaining ISC homeostasis and ensuring appropriate responses to external cues. It is noteworthy that many of the dietary nutrients mentioned above have been proven to boost ISC proliferation and self‐renewal. Therefore, supplementing these nutrients could serve as an effective therapeutic approach to promote intestinal regeneration following injury.

In recent years, the beneficial effects of calorie restriction and ketogenic diets have shown promise in managing metabolic diseases, such as obesity, insulin resistance and cardiovascular diseases. However, the observation that both calorie restriction and ketogenic diet enhance ISC proliferation and self‐renewal raises concerns. ISCs are generally considered as cells‐of‐origin for gastrointestinal cancers, linking dietary elements to elevated cancer risks. Indeed, studies have demonstrated that both HFD feeding and fast‐refeeding promote tumorigenesis in *Apc* mutant mice by enhancing ISC stemness.^60^, ^66^ Thus, it would be crucial to determine whether ISCs exhibit similar or distinct features during normal intestinal regeneration and the early stages of tumorigenesis. Moreover, investigating whether long‐term calorie restriction or ketogenic diet may promote tumorigenicity in the intestine is imperative. Nonetheless, careful consideration should be given when devising diet‐based strategies for regeneration to avoid inadvertently increasing the risk of cancer.

## AUTHOR CONTRIBUTIONS

R.S. and B.W. discussed the content, researched the data and contributed to writing the article and to reviewing and/or editing of the manuscript.

## FUNDING INFORMATION

The research in Dr. Bo Wang's lab is supported by grants from National Institute of Health (NIH) (DK114373 and DK128167) and Cancer Center at Illinois seed grant.

## CONFLICT OF INTEREST STATEMENT

The authors have no potential conflict of interest.
